# Cigarette Lighter Fluid Induced Gastric Ulcer: A Severe Complication of Delayed Foreign Body Removal

**DOI:** 10.1155/2017/4041087

**Published:** 2017-08-15

**Authors:** Ciel Harris, Lauren Stemboroski, Asim Shuja

**Affiliations:** University of Florida College of Medicine, Jacksonville, FL, USA

## Abstract

The majority of foreign bodies ingested pass uneventfully through the gastrointestinal tract without endoscopic intervention. Nevertheless, certain ingested objects pose a greater risk for complications and are more challenging to remove than others. This case report describes a 49-year-old male who swallowed a cigarette lighter causing a gastric ulcer. The lighter was successfully removed by flexible endoscopy using a polypectomy snare. Urgent removal is required due to the shape of the object and its hazardous contents. This is the first case report published in the United States describing cigarette lighter ingestion and management.

## 1. Introduction

Foreign body ingestion cases occur commonly, the majority of which result in benign spontaneous passage through the gastrointestinal tract [1–3]. Endoscopic removal is necessary in 20% of cases; however two recent studies have shown a much higher rate of intervention (63–76%) in the setting of intentional ingestion [2]. Endoscopic removal is highly successful and mortality rate is low; a study performed by Katsinelos et al. had successful endoscopic management in 98.6% of cases [3]. Foreign objects longer than 6 cm should be endoscopically removed because they may not pass through the pylorus and remain in the stomach [1]. This can lead to complications such as pressure necrosis, stricture formation, perforation, and bleeding, in which case the first-line therapy is endoscopic extraction [1, 4]. If endoscopic intervention fails, laparoscopic gastrostomy should be performed [4]. Foreign body ingestion occurs most commonly in the pediatric population [2]. Adults with psychiatric disorders, developmental delay, and alcohol intoxication and incarcerated individuals seeking secondary gain present more frequently with true foreign body ingestion [1, 2]. Edentulous adults are at greater risk of swallowing a food bolus and their dental prosthesis [2]. Ingestion of multiple objects and repeated episodes are also common [2].

## 2. Case Report

**Figure 1:**
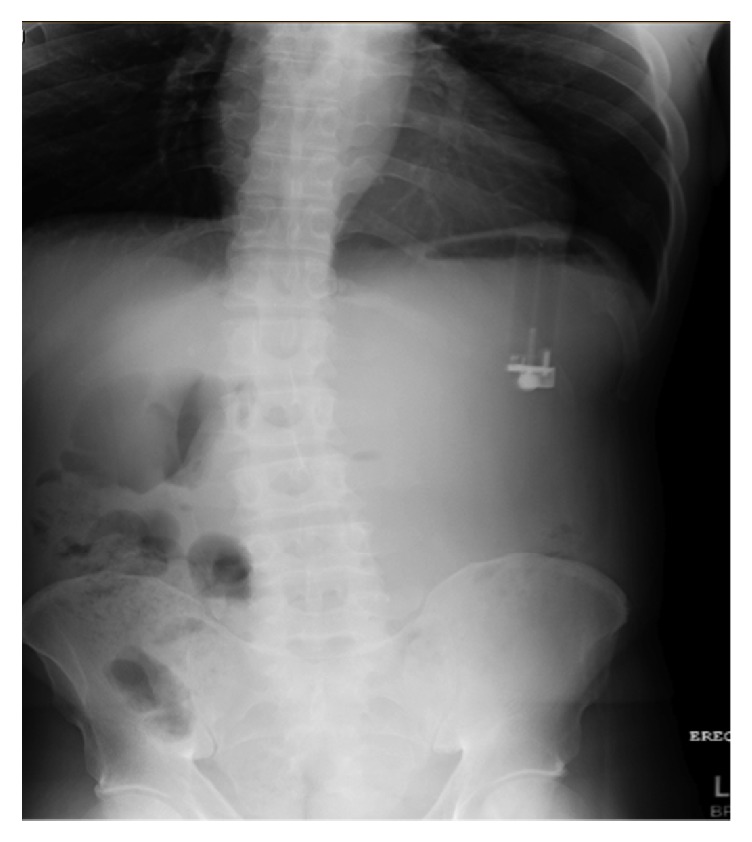
Cigarette lighter on abdominal X-ray.

A 49-year-old African American male with a history of anxiety and schizophrenia presented to the Emergency Department complaining of epigastric pain. He reported the abdominal pain as aching in nature and associated with nausea and vomiting. He endorsed a 5-pack-year smoking history. Upon chart review, he was noted to have a history of foreign body ingestion requiring laparotomy for spoon removal. Physical examination was unremarkable and his stool was guaiac negative. His laboratory investigations including blood cell counts, biochemical markers, and urinalysis were normal. Plain abdominal radiographs demonstrated a foreign body resembling a cigarette lighter visualized within the stomach (Figure 1). No abnormal air fluid levels, free intraperitoneal air, or pathologic calcifications were seen.

**Figure 2:**
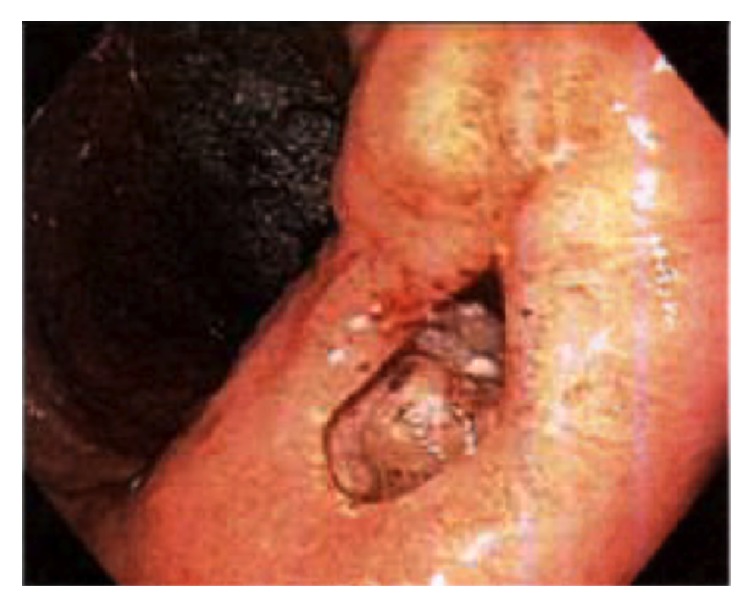
Cigarette lighter seen on endoscopy and removed via polypectomy snare.

**Figure 3:**
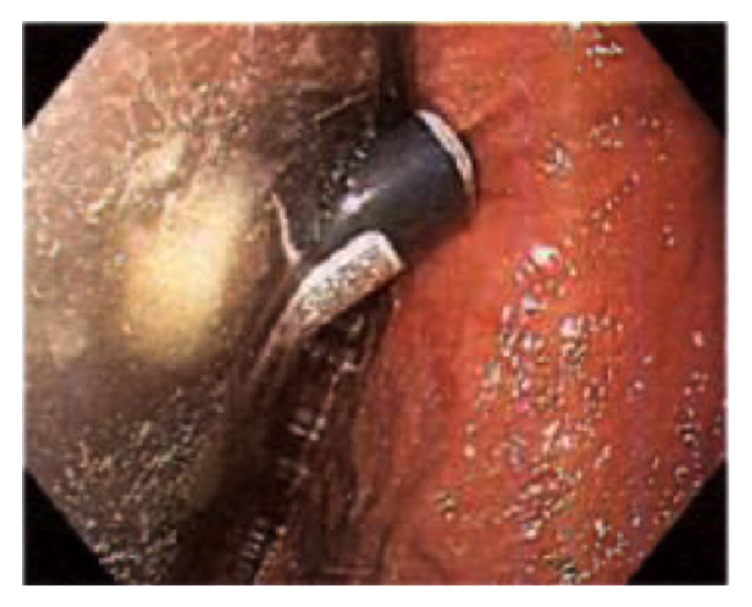
Gastric ulcer secondary to cigarette lighter fluid.

He underwent emergent upper endoscopy for foreign body removal. The examined esophagus was normal. Diffuse moderate inflammation characterized by congestion, erythema, and friability was found in the entire examined gastric mucosa. One nonbleeding cratered gastric ulcer, 5 mm in largest diameter, was found at the incisura (Figure 2). Diffuse mild duodenitis was found in the first and second part of the duodenum. A small handheld cigarette lighter was found in the gastric fundus. It was successfully removed endoscopically with polypectomy snare (Figure 3). The patient tolerated the procedure well; he was treated with pantoprazole 40 mg twice daily for eight weeks. Psychiatry consult and* H. pylori* serology was recommended. He was scheduled for a repeat endoscopy two months later to monitor treatment response.

## 3. Discussion

The incidence of lighter ingestion is not well known. Literature review yielded only 2 case reports on cigarette lighter ingestion, both of which were in other countries and both of which led to bowel obstruction. This is the only case published in the United States to our knowledge that demonstrates cigarette lighter ingestion. The patient may identify and localize the discomfort; however often it does not correlate with the site of impaction [2]. Foreign body ingestion includes a wide spectrum of clinical presentation, and the patient may be completely asymptomatic, presenting with refusal to eat, vomiting, drooling, wheezing, blood stained saliva, or respiratory distress [2]. It is also important to note that patients can remain asymptomatic for many months [1]. It is imperative that the clinician evaluate for peritonitis or small bowel obstruction [2]. Diagnosis can be made by abdominal X-rays, barium swallow, noncontrast CT scan, MRI, or direct endoscopy (flexible or rigid) if suspicion is high and imaging is unyielding [2]. Current recommendations for emergent endoscopy include complete esophageal obstruction, disk batteries, and sharp objects but not to mention cigarette lighters [2]. For successful removal the foreign body should be aligned correctly to accommodate endoscopic removal using current retrieval devices. The forward-viewing flexible panendoscope has become the instrument of choice in managing foreign bodies in most tertiary medical centers as well as in the community hospitals [5]. If this cannot be facilitated proceeding to more invasive methods of removal should be pursued. Cigarette lighters pose a unique problem given that not only its shape can lead to obstruction but also leakage of its contents can be disastrous. Harmful substances contained in lighter fluid include benzene, butane, hexamine, lacolene, naphtha, and propane [1]. The chemical properties of the individual hydrocarbon determine the specific toxicity, while the dose and route of ingestion affect which organs are exposed to the toxicity. Quite commonly the lungs are reported as most affected as the chemicals are frequently inhaled. However in our case the patient ingested the material leading to direct destruction of the stomach mucosa by the chemical irritant. Complications from this direct damage may have included perforation of the gastric wall, abscess formation, internal fistula, and hemorrhage. Indirectly hydrocarbons absorbed could lead to multisystem toxicity. In conclusion, due to this combination we recommend immediate removal of cigarette lighters and all other foreign bodies that contain hazardous material regardless of size and shape.
